# The effect of helmet materials and simulated bone and tissue layers on bullet behaviour in a gelatine model of overmatch penetrating head injury

**DOI:** 10.1007/s00414-017-1665-8

**Published:** 2017-08-16

**Authors:** Peter F. Mahoney, Debra J. Carr, David Miller, Michael Teagle

**Affiliations:** 1 Royal Centre for Defence Medicine, ICT Centre, Research Park, Birmingham, B15 2SQ UK; 2 Centre for Defence Engineering, Cranfield University, Defence Academy of the United Kingdom, Shrivenham, SN6 8LA UK; 3 Small Arms Experimental Range, Cranfield University, Defence Academy of the United Kingdom, Shrivenham, SN6 8LA UK

**Keywords:** Gelatine, Helmet, Ballistic, 7.62 × 39 mm bullet, Synthetic bone, Synthetic skin

## Abstract

The aim of this work was to simulate an overmatch ballistic event against a head wearing a helmet. The experiments were designed to understand how layers of bone (or synthetic bone), synthetic skin and currently used helmet materials influence the behaviour of full metal jacket mild steel core (FMJ MSC) 7.62 × 39 mm bullets, impacting on targets with a mean velocity of 650 m/s. Bullet behaviour within 10% (by mass) gelatine blocks was assessed by measurements made of the temporary cavity within the blocks using high-speed video and of the permanent cavity by dissecting blocks post firing. While ANOVA did not find significant difference at the 0.05 level in the mean values of most of the measurements, there was a significant difference in neck length within the gelatine blocks. The addition of material layers did produce greater variability in the temporary cavity measurements under some of the conditions. One of the synthetic bone polymers with a synthetic skin layer produced similar results within the gelatine blocks to the horse scapulae (with residual tissue) and may be suitable for future ballistic experiments.

## Introduction

Ballistic head injury remains a significant threat in combat [1]. A recent review of the open access literature [2] concluded that fatal head injuries are mainly from bullets overmatching helmets or fragments penetrating through the face. The authors also stated the need for further research into the causes and severity of head injury to assist designers of military helmets and associated personal protective equipment.

A review of gunshot injury in UK military casualties [3] looked at ballistic features associated with wound severity. The study examined extremity injuries in detail and concluded that factors associated with high energy transfer (bullets that fragmented, bullets that fractured bone and bullets that did not pass straight through the body) were associated with more complex wounds requiring repeated debridement. Factors influencing outcome from ballistic head injury are even more complicated [4] and include the volume of injured brain, overall casualty physiology (such as the presence of shock and coagulopathy) and whether the impact was from a bullet or fragment.

**Fig. 1:**
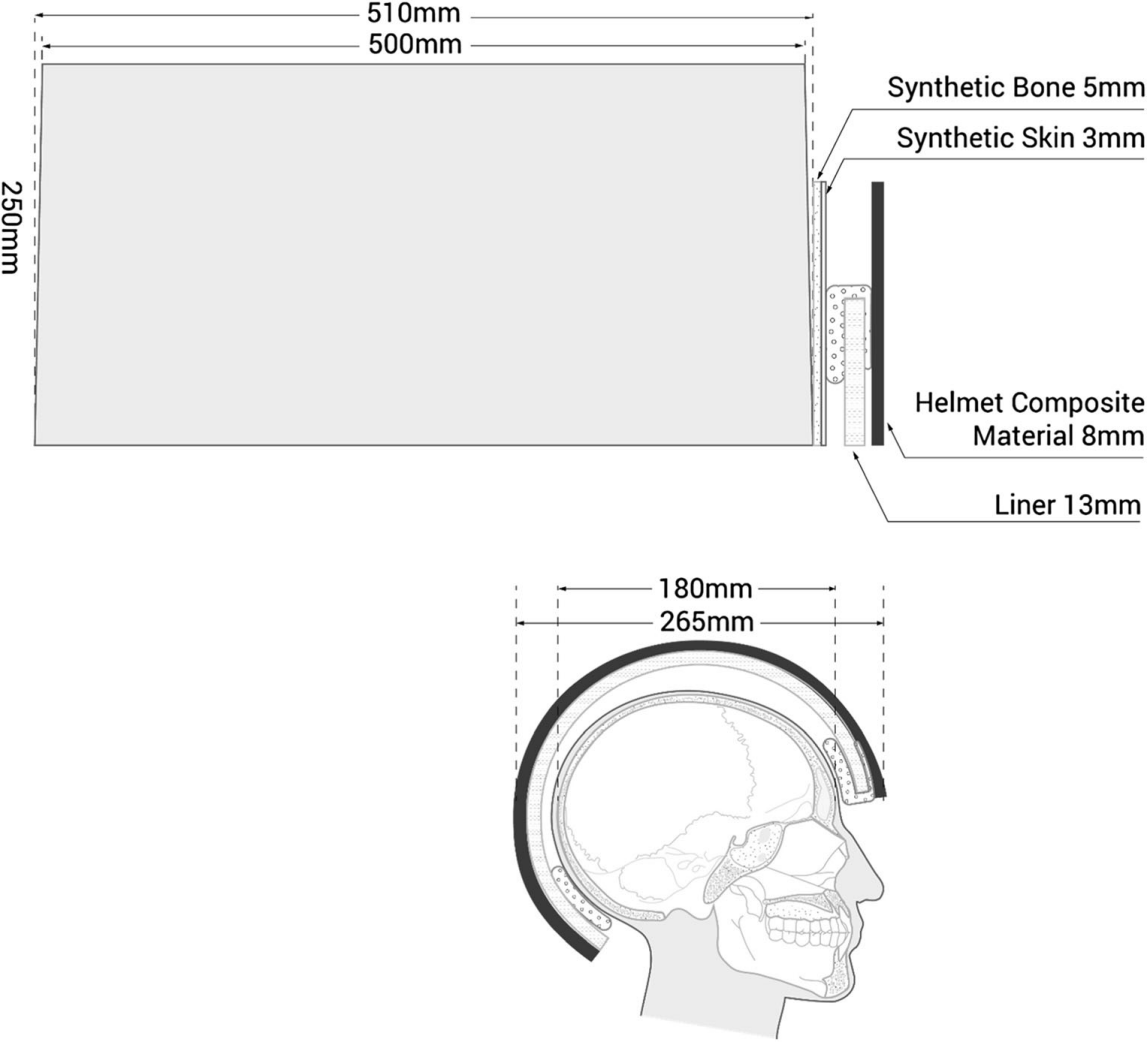
Upper image: Diagram of 10% (by mass) gelatine block and material layers placed in front of the block. Lower image: Cut away head wearing a combat helmet (on same scale as the block) to illustrate the material layers in situ. The material enclosing the top of the liner in the upper diagram is the comfort pad (seen front and rear in the lower diagram)

The aim of the work described here was to simulate an overmatch ballistic event against a simplified model of a head wearing a helmet. The experiments were designed to understand how layers of bone (or synthetic bone), synthetic skin and currently used helmet materials interact sequentially with 7.62 × 39 mm bullets fired under standard conditions and influence the bullet behaviour within 10% (by mass) gelatine blocks. The final model including all layers is summarized in Fig. 1. Understanding these interactions between the bullet and the material layers should, in turn, offer some understanding of ballistic head injury mechanisms and allow the performance of new protective materials to be assessed and compared.

A variety of approaches have been used to model ballistic injury including impacts on cadavers, animals and tissue simulants. This has been the subject of a recent review [5]. The authors describe the ethical and practical difficulties in using human materials and in vivo animal specimens for ballistic investigations. Practical issues include the variability in tissue properties among fresh, thawed and embalmed specimens [6]. Tissue simulants such as gelatine allow ballistic events to be imaged and recorded but lack the complexity of real soft tissue [5]. Our model was constructed around gelatine and synthetic materials (with the exception of horse scapulae in one of the experimental conditions) in order to standardize events as much as possible. Test materials need to be chosen with care and with an understanding of both their benefits and limitations. This will be considered further below.

### Brain

Different materials have been used to simulate brain in ballistic impact research.

Recent work by Falland-Cheung et al. [7] reviewed the properties of a selection of simulants and investigated mixtures of agar/glycerol and agar/glycerol/water (impacted with a 0.22-calibre air rifle pellet) compared with deer brain. Agar/glycerol/water specimens conditioned to 22 °C behaved in a similar fashion to the deer brain both under impact and in post impact damage patterns.

Thali et al. [8] used gelatine 10% at 4 °C to represent brain in their development of a ‘skin-skull-brain model’. The model also used a layered polyurethane sphere to represent the skull and silicone for the scalp, and the authors reported that the damage caused to the model by experimental gunshot was comparable to that seen in real injury.

Our recent work [9] has reported that synthetic skulls filled with 10% gelatine produced realistic fracture patterns when shot with 7.62 × 39 mm ammunition. No statistical difference was seen when the 10% gelatine was compared with 3, 5 and 7% gelatine and Permagel™.

Jussila [10] in describing the qualities that tissue simulants should possess, noted that they do not need to be exactly the same biomechanically as living tissue, provided ‘the results can be measured and appropriately extrapolated or scaled’.

While accepting that 10% gelatine is not a completely biofidelic brain simulant [11], its use for the current project allows reference to our earlier work [9] and the bullet behaviour to be captured by high-speed video.

Different methods have been described for assessing and evaluating the damage caused to gelatine blocks by the bullet impact.

Fackler and Malinowski [12] described four components of missile-tissue interaction (penetration, missile fragmentation, permanent cavity size and temporary cavity size). They assessed these for a series of different bullets impacting on 10% (by weight, sic) gelatine blocks and summarized them as a drawing composite to give a ‘wound profile’. They noted that the temporary cavity was largest at the point where the bullet was at maximum (90°) yaw. Berlin et al. [13] illustrate a similar observation (figure 15 of their paper) when looking at cavity size in soap blocks and relating this to bullet tumbling.

Kneubuehl [14] considers rifle bullet behaviour separately for full metal jacket and non-deforming/non-fragmenting bullets, compared with deforming and fragmenting bullets. For the type of bullet used in this current work (full metal jacket, mild steel core), he describes three distinct sections in the temporary cavity. The first section (the narrow channel or neck) is a straight entry channel. The length of this depends on the form of the bullet tip, the bullet’s gyroscopic stability and the angle of incidence at the point of impact with the target [14, p. 98].

The second section is the widest part of the temporary cavity which begins as the bullet yaws, caused by a combination of decreasing bullet velocity, increased angle of incidence within the gelatine and increased overturning moment acting on the bullet. At 90° yaw, as noted above, the bullet is in contact with the gelatine over its full length, causing rapid deceleration and energy transfer into the gelatine (Fig. 6). Rotation of the bullet about its centre of gravity forces the base or tip of the bullet into the gelatine at high velocity.

In the third section of the temporary cavity, the bullet yaws under the influence of damping forces until it is perpendicular to its direction of travel. It then tends to move forward, rocking backwards and forwards about its centre of gravity, and produces a second temporary cavity.

Fackler and Malinowski [12] estimated the diameter of the temporary cavity by dissecting the gelatine block after shooting and adding together the radial lengths of the two largest radial cracks. Subsequent work by Ragsdale and Josselson [15] using handgun ammunition fired into 20% gelatine found that these simple calculations both over and under estimated the temporary cavity when compared with measurements from high-speed films.

Jussila [16] describes how the temporary cavity and its immediate aftermath create damage within the gelatine leaving a permanent channel and fissures. This reflects the kinetic energy dissipated into the gelatine. Jussila described a number of methods to estimate this energy transfer requiring measurement of the fissures within the gelatine. Schyma and Madea [17] moulded foil bags containing acryl paint into the front of gelatine blocks such that the bullet impact spread paint all through the gelatine cracks. This in turn aided crack measurement.

Mabbot et al. [18] dissected gelatine blocks post shooting but also captured the temporary cavity using a high-speed video camera. Once the image file was calibrated using a known length visible in the picture, the pixels could be equated to millimetres. Key measurements were the largest diameter of the temporary cavity and the depth penetration of the bullets into the blocks. Our model uses 10% gelatine blocks, and the bullet impact is assessed through both images captured by high-speed video camera and post impact block dissection.

### Bone

De Boer et al. [19] measured cranial vault thickness in 1097 autopsy cases. In the adult male subgroup (655 subjects), the mean thickness of frontal bone was 6.15 mm (SD 1.91 mm)*.* The Third Patten Report [20] states that ‘a specific location on the scapula of a cow has mechanical properties similar to that of the human skull’.

This is reinforced by Smith et al. [21] who investigated the impact of flint tipped arrows on fresh cattle and pig scapulae, used to simulate human cranial bone. Smith et al. described the structural similarities as ‘areas of relatively flat bone consisting of a thin trabecular portion sandwiched between two cortical layers’ [21]. Smith also noted that the scapulae retained up to 5 mm of soft tissue and suggested this might be similar to that overlying the human cranium [21].

Bone has been simulated using a number of different polymers. While these lack the intricate structure of real bone [22], they have been shown to produce similar macroscopic fracture patterns to real bone under ballistic impact [8, 9, 23] as described above.

This current work compared impacts on flat sheets of these two types of synthetic bone and routine post mortem specimens of horse scapulae (Royal Veterinary College London). As with Smith’s work [21], the scapulae used in our work retained a layer of soft tissue of around 3 to 5 mm.

### Skin

Jussila et al. [24] undertook a review of the ballistic and mechanical properties of human skin and simulants from the published literature. They noted how the structural layers of human skin all have different properties and absorb varying amounts of impact energy and that this changes with location on the body and a person’s age. They went on to assess a series of synthetic and natural materials against published cadaveric values. Measurements included the threshold velocity required for a given projectile to penetrate the materials and the elongation at break of the materials. The best natural simulant proved to be ‘semi-finished chrome tanned upholstery “crust” cowhide’ [24]. One of the natural rubbers tested provided a possible use as a threshold velocity filter for projectile impacts but had much greater maximum elongation than human skin. The authors stated that an easy to use high fidelity synthetic material was needed for wound ballistic research.

Falland-Cheung et al. [25] have also described how factors such as age, sex and health affect the mechanical properties of human skin and how a reliable synthetic substitute would be useful for impact testing. They compared the mechanical properties of porcine skin with dental silicones. While the properties of the porcine skin and silicones differed, the silicone tear strength was similar to that reported for human skin in the literature.

For this work, synthetic skin was manufactured by Nottingham Trent University Flexural Composites Research Laboratory [NTU FCRL] and is further described below. NTU FCRL are involved in a series of projects with both the Impact and Armour Group and the Royal Centre for Defence Medicine (RCDM) simulating tissue for clinical and ballistic protection projects.

### Head model

Watkins et al. [26] illustrate the difficulties in visualizing ballistic events within the skull. They describe a model devised in the mid-1970s consisting of dried human skulls filled with 20% gelatine and covered with two layers of gelatine soaked chamois leather. They further developed this by placing a pressure transducer into the model through the foramen magnum. The models were impacted with either 3- or 6 mm ball bearings in a series of 12 experiments. In the early experiments, they used the pressure traces to understand the mechanisms occurring within the skulls during impact. In the later experiments, a pulsed X-ray source was used to produce a train of 50 images at millisecond intervals during the impact events. A cine camera was used to capture the resulting images. The cine X-ray images were then projected onto a screen and the cavities in the gelatine drawn around frame by frame for analysis. In the last two series, the pressure waves were correlated with the images.

The model used in our current work clearly does not have the morphology of a skull or a head wearing a helmet, but does represent an attempt to understand how the material layers in a head model influence bullet behaviour.

### Helmet

The design of combat helmets has evolved to defeat the ballistic and other threats of warfare [27]. Modern helmets are made of a series of discrete layers. The outer protective layer is usually a reinforced composite shell containing woven fabric. There is then a non-ballistic liner for impact protection and a size adjustment system. Comfort pads are located at the front and rear of the helmet [28]. For the model used in this experiment, para-aramid panels of the same areal density (bulk density × thickness; kg/m^2^) as an in-service helmet outer layer, the inner non-ballistic liner and a series of comfort pads were sourced from a helmet manufacturer (Morgan Advanced Materials Coventry) and the model constructed as shown in Figs. 1, 3 and 6.

Kieser et al. [29] experimented with 5.56 × 45 mm ammunition fired at deer femur embedded in 20% gelatine. They found that denim fabric draped on the anterior surface of the target caused more rapid bullet yaw, larger and more superficial temporary and permanent cavities and an increased risk of indirect fracture in the femur. A key question in our current work was whether or not the helmet materials would influence bullet behaviour and in turn impact on the ‘injury’ within the gelatine.

## Materials and methods

The research described in this paper was carried out in a number of stages (i–v below).

i. Gelatine from a single batch (GELITA® AG, UferstraBe 7, D-69412, Eberbach, Germany; Batch: 073358; Bloom strength 263) was used to manufacture 10% (by mass) gelatine blocks. The mould in which the blocks were set and conditioned measured 250 mm (w) × 250 mm (h) × 500 mm (l) producing blocks of 32 kg. The sides of the moulds tapered by 1° to facilitate set gelatine removal [18]. After setting, the blocks were conditioned at 4 °C for 24 h.

The blocks were placed 10 m down range from the end of an Enfield Number 3 Proof Housing at the Small Arms Experimental Range, Cranfield University, Defence Academy of the United Kingdom, Shrivenham.

A 5.5 mm ball bearing was fired at each block and depth of penetration measured and compared with results collected from previously published work to ensure only validated gelatine blocks were used for testing [10, 30].

Each of the six validated blocks was shot once with 7.62 × 39 mm Ukrainian mild steel core ammunition from a single batch (Soviet State Factory, Lugansk, manufactured 1967) ensuring the impact of the bullet did not overlap with the ball bearing tract. The ammunition chosen had been used in our previous work [9] and is representative of an ammunition type NATO troops have faced in recent conflict [3].

Impact velocities were recorded using a Weibel W-700 Doppler radar and the impact events recorded using Phantom V1212 and V12 high-speed video cameras set up to record the temporary cavity development within the block and the strike face impact respectively (V1212 sample rate 40,000 frames per second; exposure time 2 μs, resolution 384 × 288; V12 sample rate 28,000 frames per second; exposure time 5 μs, resolution 512 × 384).

Subsequent stages added layers in front of validated gelatine blocks into which a single projectile was fired as above.

ii. The experiment was repeated with further blocks of gelatine (*n* = 12) but with sheets of two different types of 250 mm × 250 mm × 5 mm synthetic bone placed against the block strike face (*a*. SYNBONE®, SYNBONE AG, Neugutstrasse 4, 7208 Malans, Switzerland, *n* = 6; *b*. ARRK MU51 polymer, ARRK Europe Ltd., Gloucester Technical Centre, Olympus Park, Quedgeley, Gloucester, Gloucestershire GL2 4NF, *n* = 6).

**Fig. 2:**
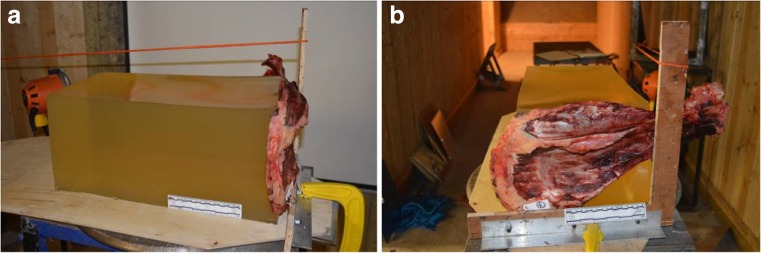
Scapulae experimental set up. **a** Scapula 6 side view. **b** Scapula 6 front view

SYNBONE® flat plates and spheres were used by Smith et al. [22] when evaluating the suitability of polyurethane bone substitutes for trauma simulations. ARRK MU51 polymer skulls were used in our recent assessment of ballistic fracture patterns in synthetic skulls [9].

iii. Horse scapulae (*n* = 6) were sourced from routine post mortem specimens (Department of Pathology and Pathogen Biology, Royal Veterinary College, London) and each was positioned in front of the strike face of a validated gelatine block. Bone thickness was measured at different sites on each scapula using calipers and a suitable impact site chosen on each (mean thickness 6.5 mm; SD 1 mm) to simulate frontal bone in line with the measurements described by De Boer and Van der Merwe [19]. The horse scapula was secured so as to ensure a flat portion was in contact with the strike face of the gelatine block (Fig. 2a). As noted above and visible in Fig. 2, a layer of soft tissue was present on the scapulae.

iv. Six sheets of synthetic skin were sourced from the NTU FCTL measuring 250 mm × 250 mm × 3 mm. This was constructed in two layers to simulate the epidermis and dermis. Both layers were made using a platinum organosiloxane gel and fibre fillers.

Each sheet was cut into three pieces. One piece of each was secured to the impact face of a sheet of MU51 synthetic bone (*n* = 6) using a two-part silicone adhesive supplied by NTU FCTL to simulate the skin and bone of the forehead. Each synthetic skin/bone assembly was placed in front of a validated gelatine block and the experiment repeated. A second piece was reserved for the experiments involving helmet layers.

The third piece of synthetic skin from each sheet was used to confirm material characteristics in accordance with BS ISO 34-1:2015 using a trouser tear test on an Instron 5567 Universal Test Machine (30 kN frame limit), computer controlled using the Bluehill 2.6 software (2005) and the load cell balanced between each test. Each specimen also underwent hardness testing with a Shore A Durometer. Characteristics of the synthetic skin are summarized in the “Results” section below.

v. Flat sheets of helmet material (250 mm × 250 mm × 8 mm), helmet liner and helmet comfort pads were purchased from a helmet manufacturer (Morgan Advanced Materials, 473 Foleshill Road, Coventry, CV6 5AQ).

**Fig. 3:**
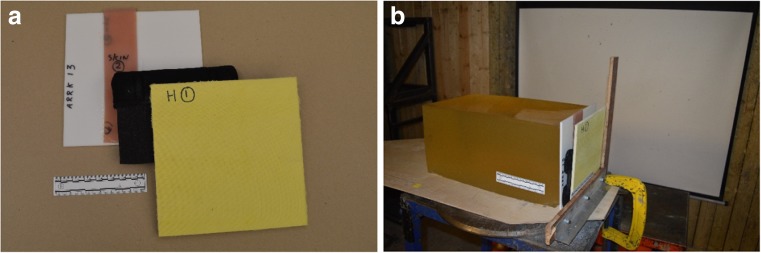
Helmet layers. **a** Layers front to back: helmet material, liner plus comfort pad, synthetic skin, synthetic bone. **b** Helmet, liner plus comfort pad and skin/bone layers in situ prior to ballistic impact

The helmet liner was cut to rectangular shapes of 200 mm × 135 mm × 13 mm to allow placement of a comfort pad (Fig. 3a).

Each layered assembly (*n* = 6) was placed in front of the same MU51 synthetic bone/synthetic skin combination described in (iv) above and both positioned in front of a validated 10% gelatine block (Fig. 3b).

The aim was to simulate a bullet perforating a military helmet and the underlying skin and bone layers before entering the brain.

### Measurements

**Fig. 4:**
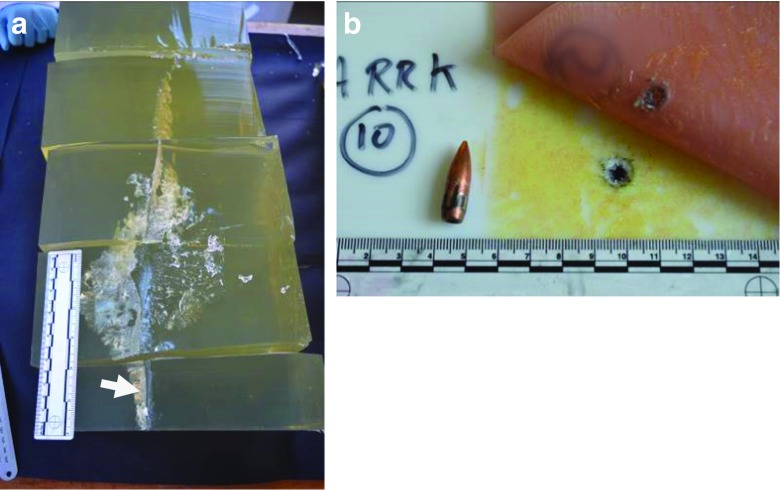
**a** Gelatine block dissection from which neck length, *n*L, (or ‘narrow channel’ [14]) was measured [arrowed]. Bullet entry is into the horizontal face at the lower aspect of the figure. Gelatine block has been cut in half lengthways to display the permanent cavity. **b** Close up of synthetic skin ‘exit wound’ and ARRK 10 ‘entry wound’ with associated bullet

Each gelatine block was dissected post firing by cutting along the permanent cavity and any debris (such as bone and polymer fragments) noted and photographed. Damaged areas within the gelatine permanent cavity were measured and photographed. The condition of the synthetic bones, horse scapulae, synthetic skin and helmet components were also photographed (e.g. Fig. 4a, b).

Measurements were taken from the high-speed video using the Phantom software (Visions Research, Phantom Camera Control Application 2.6). Each file was calibrated using a known length (forensic ruler) present in the image. As a check on the accuracy of the measurements from the images, the known lengths of the gelatin blocks and thickness of the synthetic bone plates were also measured from the images and compared with those of the actual objects and found to be within ± 0.5 mm.

**Fig. 5:**
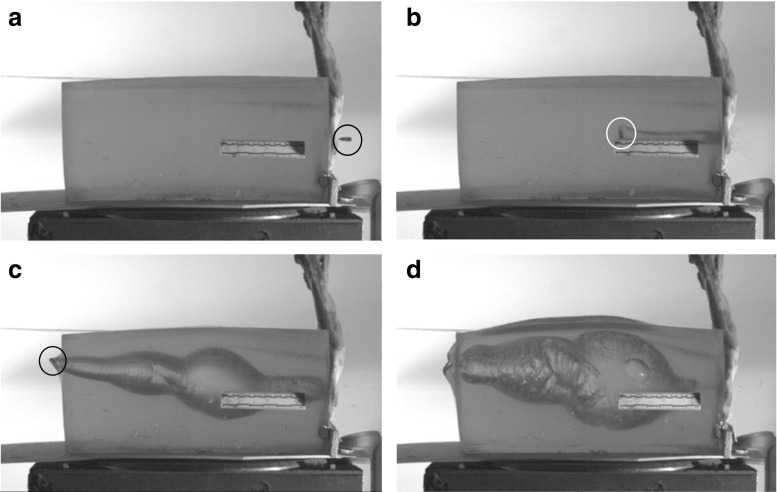
High-speed video impact sequence scapula 1, side view **a** immediately pre-bullet impact; bullet is visible in right hand side of image, **b** bullet at 90° yaw within gelatine block, **c** bullet visible on left hand side of image exiting gelatine block, **d** cavity at maximum size after bullet exit. Bullet circled in images **a**–**c**

An example impact sequence for a scapula is shown in Fig. 5a–d.

**Fig. 6:**
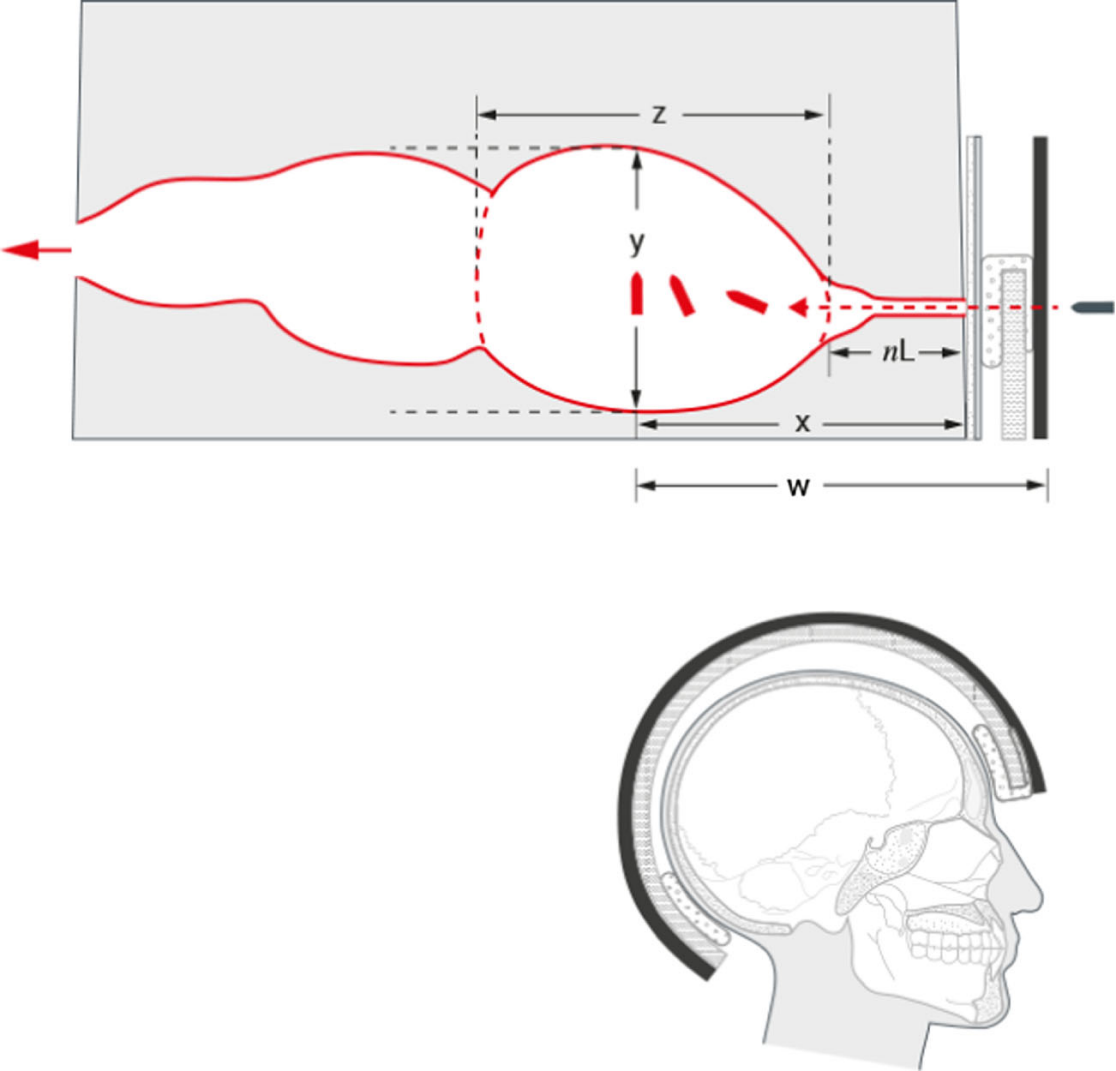
(Upper) Representation of bullet path through full model and resulting temporary cavity [after References 12, 14] with measurements taken from the high-speed video. (Lower) Head wearing helmet (to scale). Material layers and scale are as labelled in Fig. 1. w = bullet point of entry into external structures (synthetic bone, etc.) to bullet 90° yaw. x = bullet point of entry into block to 90° yaw. y = maximum height of first part of temporary cavity. z = maximum length of first part of temporary cavity. *nL* neck length; this was measured from the block dissections (Fig. 4a)

**Fig. 7:**
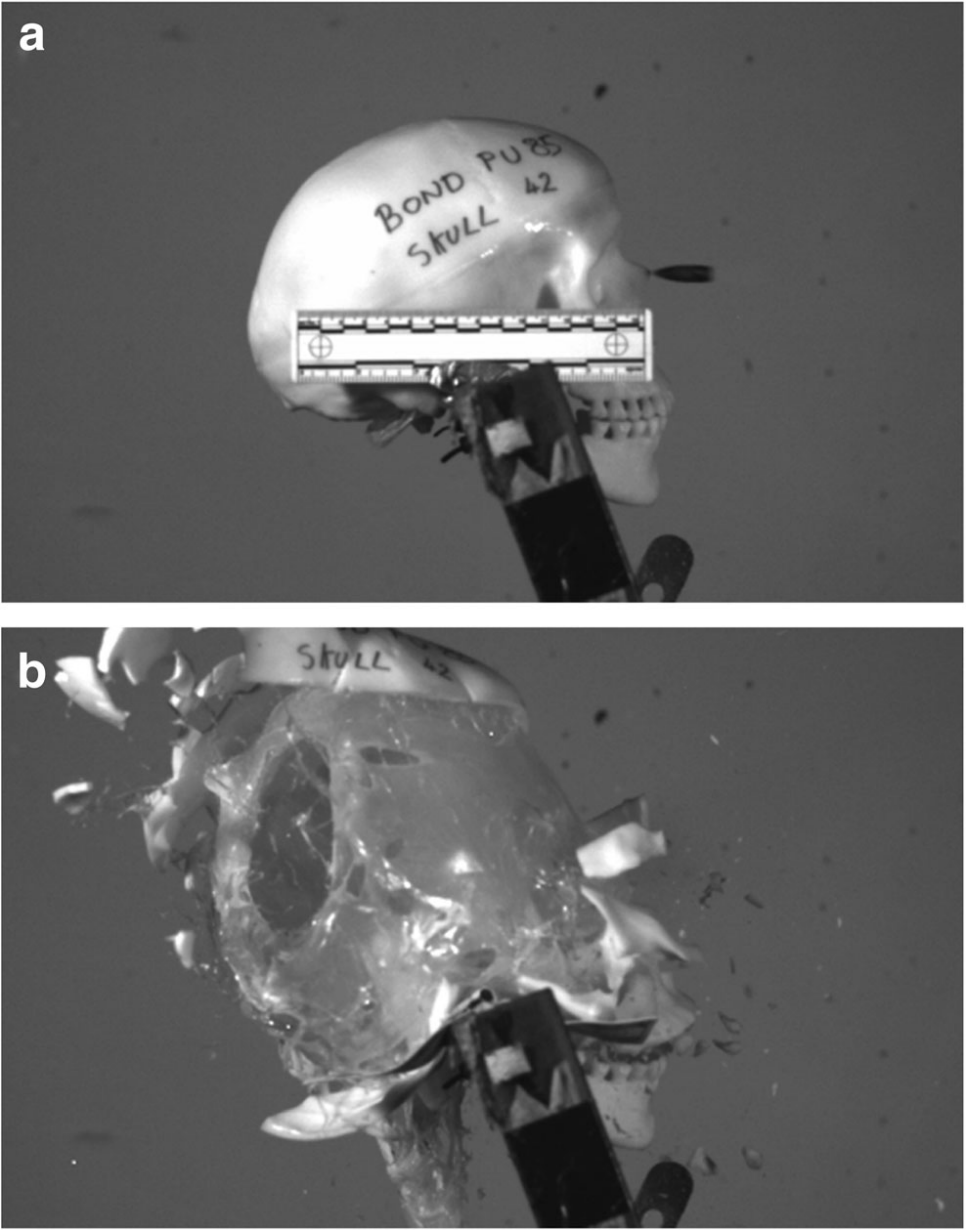
Illustration of temporary cavity in a skull model [9, 23] to show comparison with front half of the gelatine block in Figs. 6 and 8a, b. **a** Immediately pre-bullet impact. **b** Temporary cavity at maximum after bullet has passed through target. Open end of cavity in 7b is 95 mm wide; forensic scale has been torn apart by fragments and the developing cavity. Skull is same dimensions as that illustrated in Figs. 1 and 6

**Fig. 8:**
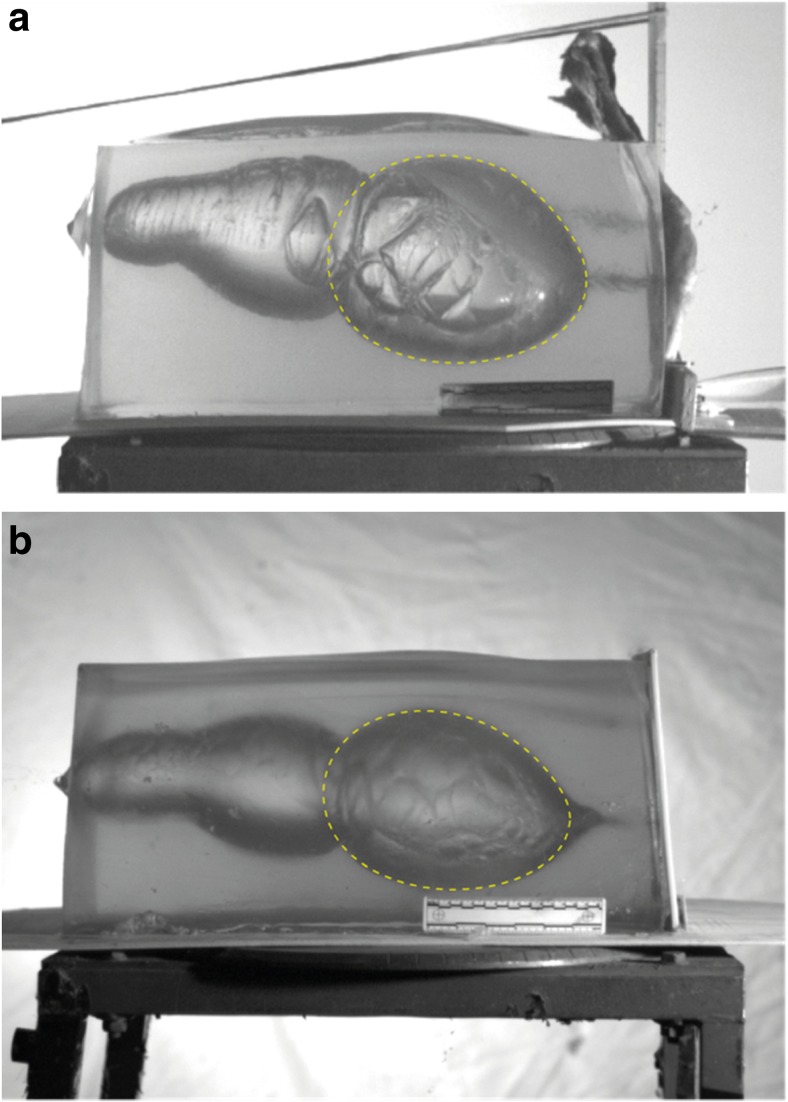
**a**, **b**. The dimensions of the first part of the temporary cavity were estimated by drawing a best fit ellipse around the cavity and estimating where the left hand border would lie (compare to Figs. 6 and 7). Gelatine blocks are same dimensions as described in Fig. 1

The area of interest for this work was the front half of the block—as the distance travelled by the bullet equates to that of a head wearing a helmet (Figs. 1, 6, 7 and 8a, b).

Tracing the cavity from a photographic image is similar to the method described by Watkins et al. [26].

The distances measured are summarized in the International Business Machines Corporation’s Statistical Package for Social Services version 24 (IBM SPSS v24) analysis section below.

The effect of external layers on distances measured in the gelatine blocks was determined using analysis of variance (ANOVA); homogeneity and normality of data was checked and a significance level of 0.05 applied. Significant differences were identified using Tukey’s honest significant difference (HSD) test.

## Results

Block temperature across all conditions was consistent (mean 7.8 °C, SD 2.3 °C) as was bullet impact velocity (mean 650 m/s, SD 9 m/s). Ball bearing impact velocity (mean 691 m/s, SD 19 m/s) and depth of penetration (DoP; mean 357 mm, SD 13 mm) was consistent with previous work [31] providing confidence within and among the groups of gelatine blocks tested.

Mean Shore hardness of the synthetic skin was measured at 21.6 DU, SD 2 DU and mean tear strength 1.76 kN/m, SD 0.35 kN/m. In comparison with Reference [25], Shore hardness of the synthetic skin was similar to reported values for human skin, pig skin and some of the dental silicones, but tear strength was lower.

The bullets passed through all the intermediate layers and perforated the gelatine blocks. Where bullets were recovered after shooting (Fig. 4b) they were intact other than some marking on the copper jacket and occasional minor deformity of the bullet tip. None of the bullets were seen to fragment on the high-speed images and no bullet fragments were recovered from the gelatine blocks.

One of the SYNBONE® sheets had cracked horizontally after the shot; all the rest appeared intact (apart from the hole from the bullet). There were no fragments of SYNBONE® material found in the gelatine blocks. Two of the six plain ARRK sheets produced plastic fragments within the permanent cavity of the corresponding gelatine blocks. Fragments were seen in the permanent tracts of all the gelatine blocks where ARRK sheets were shot with a synthetic skin layer and with the helmet material layers. None of the scapulae appeared cracked after the bullet impact, the only injury being the hole from the bullet. Bone fragments were present in five out of the six gelatine blocks from the scapula shots. Polymer and bone fragments were found between 50 to 340 mm within the gelatine blocks with no obvious link between distance and the type of intermediate layer. No helmet materials were found within the gelatine blocks.

For each of the different conditions listed the experiment was performed six times. High-speed video data was lost from one of the ARRK/skin/helmet experiments due to an onsite power failure but neck length (*n*L) data was still available from block dissection.

### IBM SPSS v24 analysis of the distances measured in the high-speed videos and block dissections (Figs. 4, 6 and 8a, b)

Measurements from the high-speed video are given in Table 1.

**Table 1:** Measurements of the distances shown in Fig. 6 for each block and material layer combination (mm)

Block number	*x* mm	*y* mm	*z* mm	*w* mm	*n*L	Material
1	183	179	201	183	110	Plain
2	175	160	213	175	100	Plain
3	225	179	228	225	100	Plain
4	196	163	228	196	100	Plain
5	173	181	233	173	90	Plain
6	184	185	233	184	80	Plain
7	231	171	232	237	140	ARRK
8	217	200	219	223	120	ARRK
9	190	175	227	197	100	ARRK
10	150	146	206	156	80	ARRK
11	182	172	208	187	130	ARRK
12	212	150	213	218	130	ARRK
13	246	196	209	253	130	SYNB
14	210	210	242	217	130	SYNB
15	213	228	240	219	164	SYNB
16	171	162	210	177	170	SYNB
17	173	164	210	180	150	SYNB
18	186	168	212	193	150	SYNB
19	192	185	205	198	110	Horse
20	218	161	201	224	110	Horse
21	238	158	212	245	140	Horse
22	140	185	213	145	30	Horse
23	180	188	229	187	120	Horse
24	168	176	208	176	110	Horse
25	161	170	210	171	60	ARRK + skin
26	124	177	218	132	70	ARRK + skin
27	189	194	221	197	80	ARRK + skin
28	216	165	225	225	120	ARRK + skin
29	221	186	246	231	150	ARRK + skin
30	181	164	210	189	80	ARRK + skin
31	130	183	227	176	30	ARRK + skin + helmet
32	Lost-power cut		−	−	45	ARRK + skin + helmet
33	126	167	205	171	40	ARRK + skin + helmet
34	104	169	203	158	50	ARRK + skin + helmet
35	185	183	238	226	60	ARRK + skin + helmet
36	188	164	216	238	100	ARRK + skin + helmet

The different materials used did not significantly affect distance *x* (bullet point of entry into block to 90° yaw), (*F*_5,29_ *=* 2.0, *p =* NS) (Table 2)*.* The SD for plain blocks (19.3 mm) was much less than for blocks with intermediate layers. The greatest SD (37.8 mm) occurred with the ARRK/skin/helmet combination. The mean value of *x* for the ARRK/skin/helmet group was different to that of the other groups, but due to the larger SD, ANOVA did not identify a statistically significant difference.

**Table 2:** Summary statistics for distance *x*—effect of intermediate materials

Material	*x* mean (mm)	SD (mm)	CV%
Plain	189.3	19.3	10.2
SYNBONE®	199.8	28.8	14.4
Horse	189.3	35.1	18.5
ARRK	197.0	29.2	14.8
ARRK + skin	182.0	36.1	19.8
ARRK + skin + helmet	146.6	37.8	25.8

Distance *y* (maximum height of first part of temporary cavity) was not affected by intermediate layers (*F*_5,29_ *=* 0.90, *p =* NS) (Table 3)*.* There was greatest variability in the SYNBONE® group followed by the plain ARRK layer. Distance *y* is controlled by the radial pressure exerted by the bullet in the gelatine block.

**Table 3:** Summary statistics for distance *y*—effect of intermediate materials

Material	*y* mean (mm)	SD (mm)	CV%
Plain	174.5	10.3	5.9
SYNBONE®	188.0	27.6	14.7
Horse	175.5	13.1	7.5
ARRK	169.0	19.5	11.5
ARRK + skin	176.0	12.0	6.8
ARRK + skin + helmet	173.2	9.1	5.3

Material did not affect the distance *z* (maximum length of first part of temporary cavity), (*F*_5,29_ = 0.6, *p* = NS) (Table 4)*.* The smallest value for *z* was for the horse bones and the largest for the plain blocks, although there was very little variability in mean or CV across all conditions.

**Table 4:** Summary statistics for distance *z*—effect of intermediate materials

Material	*z* mean (mm)	SD (mm)	CV%
Plain	222.7	12.9	5.8
SYNBONE®	220.5	15.9	7.2
Horse	211.3	9.7	4.6
ARRK	217.5	10.4	4.8
ARRK + skin	221.7	13.3	6.0
ARRK + skin + helmet	217.8	14.8	6.8

Distance *w* (bullet point of entry to external structures to bullet 90° yaw) did not vary significantly among block groups (*F*_5,29_ = 0.3, *p* = NS) (Table 5). Plain gelatine was less variable than the blocks with intermediate layers.

**Table 5:** Summary statistics for distance *w*—effect of intermediate materials

Material	*w* mean (mm)	SD (mm)	CV%
Plain^a^	189.3	19.3	10.2
SYNBONE®	206.5	28.9	14.0
Horse	195.8	35.4	18.1
ARRK	203.0	29.3	14.4
ARRK + skin	190.8	36.5	19.1
ARRK + skin + helmet	193.8	35.7	18.4

^a^The values for distance *x* and *w* for plain blocks are identical as there are no additional layers

Neck length (*n*L) was affected by intermediate layers (*F*_5,29_ = 7.30, *p* ≤ 0.001) (Table 6). Tukey’s HSD produced three overlapping groups:

- Group 1 (plain, horse, ARRK + skin, ARRK + skin + helmet)
- Group 2 (plain, horse, ARRK, ARRK + skin)
- Group 3 (horse, ARRK, SYNBONE®)

**Table 6:** Summary statistics for ‘*n*L’—effect of intermediate materials

Material	‘*n*L’ mean (mm)	SD (mm)	CV%
Plain	96.7	10.3	10.6
SYNBONE®	149.0	16.7	11.2
Horse	103.3	37.8	36.6
ARRK	116.7	22.5	19.3
ARRK + skin	93.3	34.4	36.9
ARRK + skin + helmet	56.0	27.0	48.2

This indicates that *n*L in the full model of ARRK + skin + helmet is different to that with SYNBONE® as the intermediate layer.

In addition to the ANOVA a number of other observations can be made. With measurement *x* (bullet yaw to 90°), there is greater variability as the model becomes more complex. There is an effect of the external layers on distance *w* (distance to bullet yaw to 90° taking into account the external layers) but there is overlap across the different conditions. With distance *y* (temporary cavity height), there was the greatest variability with the SYNBONE® and plain ARRK sheets, but less with the horse, ARRK + skin and the full helmet model. With neck length (*n*L), there was the greatest variation with the horse, ARRK + skin and full helmet model.

The horse and ARRK plus skin produced very similar results for distances *w*, *x*, *y* and ‘*n*L’.

## Discussion

Ballistic head injury is complex and outcome is influenced by many factors [1–4]. Wearing military helmets is associated with reduced fatalities from ballistic impact [2]. Mechanisms include projectile deflection and energy dissipation by the helmet materials, although above a particular impact energy, the helmet materials will be defeated.

The aim of this work was to simulate an overmatch ballistic event against a simplified model of a head wearing a helmet and understand how the intermediate layers of material influence the behaviour of FMJ MSC 7.62 × 39 mm bullets. The main findings were that increased complexity in the model (i.e. additional layers) increased the variability (1) in distance from impact on the surface of the block to 90° yaw of the bullet (distance *x*) and (2) in neck length/narrow channel length within the gelatine block.

As noted above, Kneubuehl has described how the neck length depends on the form of the bullet tip, the bullet’s gyroscopic stability and the angle of incidence at the point of impact with the target [14, p. 98]. The experiment reported in the current paper controlled for bullet tip variation by using rounds from the same manufactured batch. The angle of incidence was controlled as far as practical under the experimental conditions but as seen in Figs. 3b, 5a, b and 8a, b, there are very small differences in the impact angles presented by different targets.

On the high-speed video and at block dissection the initial bullet path (i.e. the neck) within all the gelatine blocks appeared horizontal after passing through the intermediate layers; thus, intermediate layers did not affect bullet directionality along the horizontal centre axis. However, the results in Table 6 suggested that intermediate layers influenced gyroscopic stability, i.e. intermediate layers appeared to affect the propensity of the bullet to start yawing.

The effects in our model are less clear cut than those described by Kieser et al. [29] (described in the helmet section of the introduction) where denim fabric draped on the gelatine impact surface caused 5.56 × 45 mm bullets to yaw more rapidly, produce larger cavities and increase the risk of indirect fractures in the deer femur embedded in gelatine. The bullets used by Kieser et al. [29] tended to fragment within the gelatine blocks. This does illustrate how such interactions will vary with the bullet characteristics and material types. Even with the plain gelatine blocks without intermediate layers, there was variation in the temporary cavity measurements (as indicated by the CVs), despite factors such as bullet type, impact velocity, impact site on the gelatine, gelatine concentration and consistency, and temperature being controlled for. This supports Kneubuehl’s view of the empirical nature of wound ballistics [14, p. 87].

Additional work is required to understand further how bullet interactions with helmet materials at overmatch influence wound profiles and how this relates to resulting clinical injury.

In terms of bullet damage, the scapulae and synthetic bone behaved in a similar fashion. For most targets, the only damage seen was the bullet hole, although one of the SYNBONE® sheets had cracked horizontally. The explosive effect illustrated in Fig. 7 is a feature of the rapid rise in intracranial pressure from the temporary cavity within a filled skull model and is described further in References [9, 23].

Previous work has been undertaken to find suitable synthetic tissue substitutes for the skin [8, 24, 25] and bone [8, 9, 22, 23], so it is reassuring to find that the results for ARRK + synthetic skin were very similar to those for the scapulae (with residual tissue layer) across a number of the measurements.

## Conclusions

Using FMJ MSC 7.62 × 39 mm bullets, there was an effect on neck length within the gelatine blocks when intermediate material layers were perforated suggesting an influence on bullet gyroscopic stability. Variability was observed in measurements within each experimental condition. The addition of material layers produced greater variability in the temporary cavity measurements under some of the conditions. Typically, variability increased with increasing complexity of the intermediate layers. One of the synthetic bone polymers with a synthetic skin layer produced similar results within the gelatine blocks to the horse scapulae (with residual tissue) and may be suitable for future ballistic experiments.

### Limitations of the model

This model only used one type of ammunition at velocities chosen to overmatch the helmet materials. Different results might be obtained across a range of velocities and with alternative ammunition types.
